# Management of penetrating abdominal trauma in St Mary's Hospital Major Trauma Centre

**DOI:** 10.1186/1757-7241-23-S2-A9

**Published:** 2015-09-11

**Authors:** Rachael C Fisher, Carina McGuire, Christopher J Aylwin

**Affiliations:** 1 Major Trauma Ward, St Mary's Hospital, London, UK

## Background

There continues to be a debate into the management of penetrating abdominal injury. The use of CT has improved the safety of conservative management and laparoscopy in trauma has been postulated as an alternative to trauma laparotomy [1].

## Method

A retrospective review was undertaken of all patients admitted as a trauma call to St Mary's Hospital between January 1^st^ 2012 to 30^th^ April 2014 with penetrating abdominal trauma. Information was obtained regarding their mechanism of injury and management through the A+E symphony database, PACS system and the discharge letter database.

## Results

145 patients were identified, 135 males and 10 females, average age 29. Stabbing was the mechanism of injury most frequently accounting for 77% (111/145) of presentations. In total 36% of injuries were managed conservatively, with 64% undergoing operative management.

The median length of stay for isolated abdominal injuries, irrespective of pathology found, ranged from 1 day in those conservatively managed; 1.5 days in those undergoing laparoscopy; to 6 days for those undergoing a laparotomy.

In patients who had negative intraabdominal pathology the median length of stay was 1 day for both conservatively managed and laparoscoped patients compared to 2 days if they underwent an exploratory laparotomy. Notably 50% of all laparoscoped patients had negative operative findings.

## Discussion

Our data shows laparoscopy as a safe alternative to laparotomy in penetrating abdominal trauma, halving the median length of stay. Although laparosocopy has a lower risk profile, 50% of laproscoped patients had negative findings, potentially suitable for conservative management.

Laparosocopy may be an option for equivocal findings, however the combination of advancing imaging techniques and accuracy of reporting suggest operative management could be reserved for haemodynamically unstable and radiologically proven injuries.
